# Effects of Seasonings on the Stable Isotope Analysis of Protein Fractions in Cooked Beef: A Preliminary Study for Geographical Origin Purposes

**DOI:** 10.3390/foods15010012

**Published:** 2025-12-19

**Authors:** Yaeko Suzuki, Rie Satoh, Ayano Watanabe, Mifumi Morita, Yasuharu Takashima

**Affiliations:** 1 National Agriculture and Food Research Organization (NARO), Tsukuba 305-8642, Ibaraki, Japan; 2 Institute of Food Research, National Agriculture and Food Research Organization (NARO), Tsukuba 305-8642, Ibaraki, Japan; satoh.rie326@naro.go.jp; 3 Food and Agricultural Materials Inspection Center (FAMIC), Saitama 330-9731, Saitama-shi, Japan; ayano_watanabe527@famic.go.jp (A.W.); mifumi_morita711@famic.go.jp (M.M.); yasuharu_takashima468@famic.go.jp (Y.T.)

**Keywords:** beef, cooked beef products, geographical origin, stable isotope ratio, protein

## Abstract

This study focused on proteins derived from beef to minimize the influence of seasonings when developing a method for determining the geographical origin of seasoned beef samples. The seasoning used was sweetened soy sauce containing sugar, soy sauce, mirin and sake. The water-soluble fraction was extracted as a cleaning step for the sample, followed by extraction of the myofibrillar protein fraction. No significant differences were observed in the carbon, nitrogen and oxygen isotope ratios of the proteins extracted from the defatted raw and cooked beef samples. The carbon, nitrogen and oxygen isotope ratios of the protein fraction extracted from defatted beef were positively correlated with the corresponding ratios in the defatted whole beef samples. These results suggest that the protein fractions were mainly composed of beef proteins, and that the addition of auxiliary materials did not affect this. To verify the possibility of determining the geographic origin of beef, the carbon, nitrogen and oxygen isotope ratios of proteins extracted from beef from the United States (U.S.), Australia and Japan were analyzed. The carbon isotope ratios of proteins extracted from U.S. beef were higher than those of Australian and Japanese beef. Additionally, the oxygen isotope ratios of proteins extracted from Australian beef were higher than those of beef from the U.S. and Japan. These results suggest that it may be possible to trace the geographical origin of beef products cooked with seasonings by extracting proteins.

## 1. Introduction

Beef is one of the commodities that Japan relies on imports. In 2024, beef production amounted to 353,435 tons, while beef imports reached 506,260 tons [1]. On the other hand, Japanese beef, especially Wagyu, is in high global demand because of its high quality and as market access for Japanese beef exports is increasing. Japan’s beef exports in 2024 reached 10,113 tons, which was a significant increase of 20.1% from the previous year, and the highest volume on record. Exports were made to 51 countries and export value reached $420 million, a large increase of 11.6% over the previous year, also the highest on record [2].

Beef can be processed into a variety of food products, such as hamburgers, roast beef, minced cutlets, croquettes, beef jerky, corned beef, curries, stews, and marinades for grilled meat. These products can be enjoyed as convenient, ready-made meals at home or given as gifts, providing an easy way to enjoy beef. Products made with premium beef, such as Wagyu, may have labels emphasizing the country of origin or the use of geographical indications (GIs). In Japan, the food labeling standards were revised in 2017 to make it mandatory to label the origin of the main ingredients of all processed foods. This rule has been fully enforced since April 2022 [3]. There is a need to develop technology to identify the place of origin of cooked beef products.

Many studies have been reported on techniques for determining the geographical origin of beef using DNA analysis and stable isotope ratio analyses [4,5,6,7,8,9,10,11,12]. However, these studies were based on fresh meat, and there are few reports on determining the geographical origin of cooked food products. The advantage of stable isotope ratio analysis is that it is less affected by cooking. Zhou et al. (2015) reported that cooking processes such as grilling and boiling had a small effect on the carbon and nitrogen isotope ratios in beef [13]. There are no reports on origin identification techniques for processed beef products including secondary ingredients.

Since auxiliary ingredients and seasonings also contain carbon, nitrogen and oxygen isotope ratios, it is necessary to reduce the effects of these ratios. Suzuki et al. 2021 focused on wheat-derived proteins in bread and reported that the origin of wheat could be determined by analyzing the stable isotope ratios of the extracted protein fraction [14]. Depending on solubility differences, water-soluble (albumin/globulin), alcohol-soluble (gliadin) and SDS-soluble (glutenin) fractions were extracted sequentially to verify differences in each protein fraction based on the presence or absence of auxiliary ingredients. The glutenin fraction showed no differences in electrophoretic patterns or stable isotope ratios, suggesting that it could be used to identify the geographical origin of wheat flour in bread.

The seasonings used to prepare the beef in this study, such as sugar, soy sauce, mirin and sake, are commonly employed in Japanese cuisine. These seasonings contain low levels of protein. Thus, this study focused on proteins derived from beef. Generally, meat consists of approximately 70% water, around 20% muscle protein and the rest is made up of lipids, carbohydrates and vitamins [15]. Muscle proteins are classified by solubility as sarcoplasmic (water-soluble), myofibrillar (salt-soluble) or stromal (insoluble) [15,16]. As the main components in seasonings are water-soluble, myofibrillar proteins were targeted.

## 2. Materials and Methods

### 2.1. Cooking Beef to Evaluate the Effect of the Presence or Absence of Auxiliary Materials Using Stable Isotope Analysis

One Australian beef sample was purchased from a market to prepare the cooked beef samples. The boiled beef was prepared by boiling the raw beef in boiling water for five minutes. The marinated beef consisted of 200 g of fresh beef soaked overnight in a marinade made with 45 mL of soy sauce, 6 g of sugar, 15 mL of sweet cooking rice wine (mirin), and 15 mL of rice wine (sake). The simmered beef was prepared by placing the marinated beef and marinade in a pot and simmering over a medium heat for around 10 min.

### 2.2. Beef Samples for Tracing Their Geographical Origin

We collected 32 raw beef samples, which were used to trace their geographical origin: nine from Japan, 15 from the U.S., and eight from Australia. Of the Japanese samples, four were Black Japanese, three were crossbreeds, and two were Holsteins. All of the beef from Australia and the U.S. was Angus.

### 2.3. Extraction of Protein Fractions

Compared to other tissue components such as proteins, lipids have lower carbon isotope ratios. Therefore, when the lipid content of a sample is high, the carbon isotope ratio of the entire sample will be lower. To eliminate this lipid-induced bias, all raw and cooked beef samples were defatted using a chloroform–methanol solution (2:1, *v*/*v*) and ground in a laboratory mixer, as previously described [5].

The defatted samples (50 mg into a 2.0 mL microtube and mixed with 1.25 mL of 50 mM Tris–HCl buffer (pH 8.8) for 10 min using an ultrasonic device on ice (MCD-10, AS ONE Corp., Osaka, Japan) as described previously [14]. To remove the supernatants (albumin/globulin fraction), the mixtures were centrifuged at 20,000 *g*, for 10 min at 4 °C. The pellets were stirred in 1.25 mL of 50 mM Tris-HCl buffer (pH 8.8) and washed once for 10 min using an ultrasonic device on ice. They were centrifuged at 20,000 *g* at 4 °C. for 10 min and removed the supernatants. The pellets were stirred in 1.25 mL of 8 M urea containing 2 M thiourea and 4% (*w*/*v*) CHAPS((3-(3-Cholamidopropyl)dimethylammonio)-1-propanesulfonate)) for 10 min using an ultrasonic device on ice as previously described for the extraction of myofibrillar proteins [16]. They were again centrifuged at 20,000 *g* at 4 °C. for 10 min and removed the supernatants. The supernatants were collected as protein fractions. The protein components were precipitated by adding a 10-fold amount of pre-cooled acetone to the supernatants. After vortexing, the mixtures were incubated at −80 °C for 60 min. They were centrifuged at 20,000 *g* at 4 °C for 10 min. The obtained pellets were washed with ultrapure water and ethanol and air-dried in a draft and used for measuring the δ^13^C, δ^15^N and δ^18^O values. This extraction method was performed three times on each sample.

### 2.4. Stable Isotope Analysis

The carbon, nitrogen and oxygen isotope ratios were analyzed according to the procedure described by Suzuki et al. 2021 [14]. In brief, for the analysis of carbon and nitrogen isotopes, approximately 1.0 mg of defatted raw or cooked beef sample was weighed into a tin capsule (5.0 × 9.0 mm), as was approximately 0.3 mg of the air-dried protein fraction. The measured δ^13^C and δ^15^N values were normalized by using five isotope-known amino acid standards (Histidine: δ^15^N = −7.6‰ and δ^13^C = −10.1‰; L-Alanine: δ^15^N = −1.06‰ and δ^13^C = −19.94‰; Glycine: δ^15^N = +1.2‰ and δ^13^C = −30.5‰; L-Alanine: δ^15^N = +10.1‰ and δ^13^C = −19.6‰; and L-Alanine: δ^15^N = +20.0‰ and δ^13^C = −19.6‰), which were purchased from Shoko Science Co., Ltd., Japan. These standards were calibrated by using the Dual-inlet method with the following international standards: IAEA-N-1, IAEA-N-2 and NBS 19-limestone (NIST RM #8544). Five working standards were determined and analyzed for every twelve samples to confirm the reproducibility of the measurements. For the oxygen isotope analysis, approximately 1.0 mg of defatted raw or cooked beef sample, as well as air-dried protein fractions, were weighed into silver capsules (3.3 × 5.0 mm). The individual samples were then analyzed using an elemental analyzer/isotope ratio mass spectrometer (EA/IRMS) via IsoPrime 100 (Isoprime Ltd., Manchester, UK) interfaced with an Elementar Vario PYRO cube (Elementar Analysensysteme GmbH, Langelselbold, Germany). The measured δ^18^O values were then normalized using isotopically known benzoic acid standards (+71.4‰ and +23.2‰), which were purchased from Indiana University (the U.S.). Four working standards were determined for every twelve samples to confirm the reproducibility of the measurements: dibenzo-24-crown-8 (−15.7‰), dibenzo-18-crown-6 (+1.7‰), β-D-galactose pentaacetate (+12.7‰), and D-(+)-sucrose octaacetate (+26.8‰). The standard deviations of the three replicates were less than 0.2‰ for δ^13^C and δ^15^N and 0.5‰ for δ^18^O values.

The isotopic composition was reported in the δ notation:

𝛿 = (R sample/R standard − 1)(1)

where R is the isotope ratio (i.e., ^13^C/^12^C, ^15^N/^14^N and ^18^O/^16^O) of the sample, and R standard is the isotope ratio of the international standards (for carbon: Vienna PeeDee Belemnite, for nitrogen: Air, and for oxygen: Vienna Standard Mean Ocean Water). Isotope values are indicated per mil (‰).

### 2.5. Statistical Analysis

A paired *t*-test was performed to determine whether there were significant differences in the δ^13^C, δ^15^N and δ^18^O values of the beef samples and protein fractions in the defatted raw and cooked beef samples. Pearson product-moment correlation coefficients were used to independently test the correlation between the δ^13^C, δ^15^N and δ^18^O values of the raw beef and protein fractions independently. A one-way analysis of variance (ANOVA) was performed to evaluate the significance of the differences in the δ^13^C, δ^15^N and δ^18^O values of protein fractions obtained from beef sourced in three countries: Japan, the U.S., and Australia. Statistical significance was set at *p* < 0.05. Principal component analysis (PCA) was performed using R software (version 4.5.1, R Development Core Team, 2025) to characterize the beef samples from the three countries. A 95% confidence ellipse was drawn using R software and the ggplot2 package (version 4.0.0) [17].

## 3. Results

### 3.1. Effects of Auxiliary Materials on δ^13^C, δ^15^N and δ^18^O Values of Protein Fractions

The mean and 1 σ standard deviation of the δ^13^C, δ^15^N and δ^18^O values of defatted whole raw beef, defatted whole cooked beef, and their protein fractions are shown in Table 1. The values for the protein fractions in Table 1 were the means and standard deviations of measurements taken from three separate extractions of each sample. The δ^13^C, δ^15^N and δ^18^O values between the defatted whole raw beef and defatted whole cooked samples were significantly different (*p* < 0.05). The δ^13^C and δ^18^O values of the defatted whole marinated, and simmered beef were increased compared to those of the defatted whole raw beef.

There is no significant difference in the δ^13^C and δ^18^O values between the protein fractions of the defatted raw beef and its cooked products (*p* = 0.13–0.85 in δ^13^C values, *p* = 0.12–0.94 in δ^15^N values and *p* = 0.36–0.65 in δ^18^O values). These results suggest that the protein fractions consist primarily of beef protein and are not affected by the seasoning.

**Figure 1:**
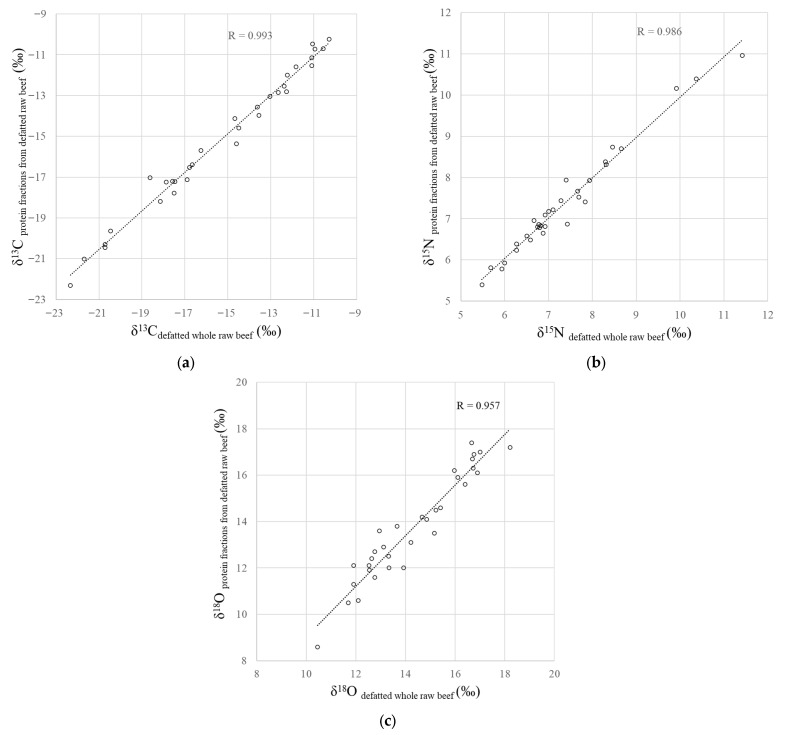
Relationship between the (**a**) δ^13^C, (**b**) δ^15^N and (**c**) δ^18^O values of defatted whole raw beef samples and the protein fractions obtained from defatted raw beef samples.

The δ^13^C, δ^15^N and δ^18^O values of the protein fractions extracted the defatted whole beef samples were positively correlated with those of the defatted whole raw beef (Figure 1, R = 0.993, *p* < 0.001 for δ^13^C; R = 0.986, *p* < 0.001 for δ^15^N; R = 0.957, and *p* < 0.001 for δ^18^O). These results demonstrate that the protein fraction extracted from defatted beef samples using a myofibrillar protein extraction solution consists primarily of proteins originating from the beef samples themselves.

### 3.2. δ^13^C, δ^15^N and δ^18^O Values of the Extracted Proteins from Japanese, the U.S., and Australian Beef

**Figure 2:**
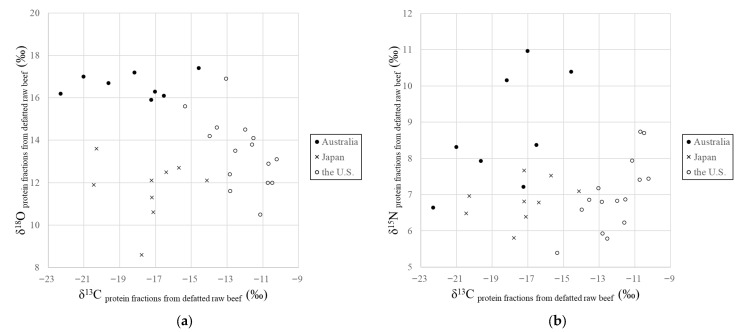
(**a**)The δ^13^C and δ^18^O values, and (**b**) the δ^13^C and δ^15^N values of the protein fractions obtained from defatted beef samples from Japan, the U.S., and Australia.

The distributions of the δ^13^C, δ^15^N and δ^18^O values of the extracted proteins from Japanese, the U.S., and Australian beef samples are shown in Figure 2. The δ^13^C values of the U.S. beef (−12.0 ± 1.3‰, mean; ±1 σ, standard deviation) were higher than those of Japanese beef (−17.6 ± 2.0‰) and Australian beef (−18.3 ± 2.9‰; *p* < 0.001). The δ^18^O values of Australian beef (16.7 ± 0.6‰) were higher than those of Japanese beef (12.3 ± 0.8‰) and the U.S. beef (14.2 ± 1.4‰; *p* < 0.001). The δ^15^N values of Australian beef (+8.8 ± 1.6‰) were relatively higher than those of Japanese beef (+6.8 ± 0.6‰) and the U.S. beef (+7.0 ± 0.9‰; *p* < 0.001). These results suggest that the geographical origin of seasoned beef products can be determined by extracting proteins.

**Figure 3:**
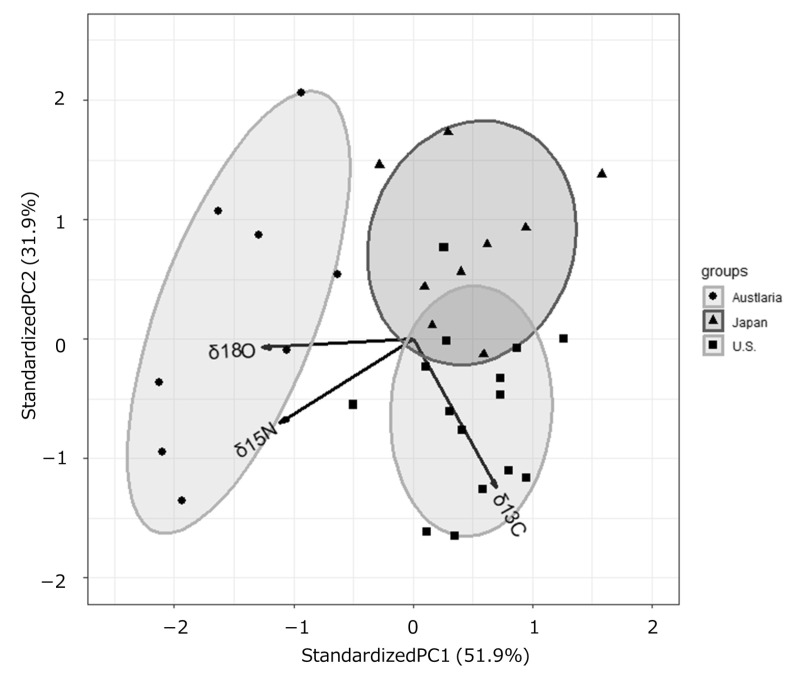
Principal component analysis of the δ^13^C, δ^15^N, and δ^18^O values derived from the protein fractions obtained from defatted beef samples from Australia, Japan and the U.S.

The results of PCA using the δ^13^C, δ^15^N, and δ^18^O values of the protein fractions obtained from defatted beef samples were shown in Figure 3. Samples with higher δ^15^N and δ^18^O values had lower PC1 scores, while samples with higher δ^13^C values had higher PC1 scores and lower PC2 scores. PC1 and PC2 together accounted for 83.8% of the total variation, and the factor loadings also showed the characteristics of the three countries in the three variables.

## 4. Discussion

### 4.1. Effects of Auxiliary Materials on δ^13^C, δ^15^N and δ^18^O Values of Protein Fractions Effects of Auxiliary Materials on δ^13^C, δ^15^N and δ^18^O Values of Protein Fractions

The carbon isotope ratios of the whole defatted marinated and simmered beef were higher than those of the raw beef samples. Bostic et al. 2015 reported that the δ^13^C and δ^15^N values of grain-based foods do not change during cooking processes such as baking or fermentation and can be estimated from the isotopic composition of the raw materials used [18]. Using representative values for most raw ingredients, the δ^13^C values of processed or cooked foods can be reasonably estimated. However, cookies baked using beet sugar or cane sugar show significantly different carbon isotope ratios, indicating that the type of sugar used can alter the δ^13^C value of cooked foods. In this study, sugar was also included in the seasoning for both the marinated and simmered beef. Globally, approximately 80% of sugar production comes from cane sugar, while around 20% comes from beet sugar. Cane sugar is derived from C_4_ plants, which have a high carbon isotope ratio. Therefore, to analyze the stable isotope ratios in processed foods, the influence of sugar needs to be removed.

The results for the whole defatted samples showed that the nitrogen isotope ratios were relatively less affected by seasonings, compared to the carbon and oxygen stable isotope ratios. On the other hand, the whole defatted boiled and simmered beef samples had a tendency toward lower nitrogen isotope ratios. This variation may be due to the thermal decomposition and loss of amino acids and peptides during heating process. Royer et al. 2017 cooked various animal meats and fish using three methods (boiling, frying, and barbecuing) and reported changes in the carbon, nitrogen, and oxygen isotope ratios caused by cooking [19]. They showed that δ^13^C, δ^15^N, and δ^18^O isotope ratios were shifted by up to 1.8‰, 3.5‰, and 5.2‰, respectively, during the cooking process. These shifts are likely related to biochemical reactions occurring during cooking, such as dehydration and the thermal decomposition of lipids, amino acids, and peptides. This suggests that heating can alter the isotopic composition of animal tissues under specific conditions.

Therefore, based on prior research, stable isotope ratios may change during the cooking process when animal ingredients constitute the majority of carbon, nitrogen or oxygen in a food product or when cooking methods involving high sugar content are used. When analyzing such foods, it is necessary to analyze these ingredients separately.

Suzuki et al. 2021 reported on the potential for developing a technique to identify the origin of wheat flour in bread [14]. Focusing on wheat-derived proteins, they analyzed the stable isotope ratios of extracted protein fractions with the aim of developing a technique to identify the origin of raw wheat in wheat processed products. To evaluate the effect of the presence or absence of secondary ingredients in protein extraction, bread was baked using a base of wheat flour, sugar, and salt, with the addition of yeast, skim milk, or butter. From each sample, water-soluble fractions (albumin/globulin fraction), alcohol-soluble fractions (gliadin fraction), and SDS-soluble fractions (gluten fraction) were extracted sequentially according to their solubility. SDS-polyacrylamide gel electrophoresis (SDS-PAGE) was used to verify differences in each protein fraction with and without secondary ingredients. The glutenin fraction, which showed no differences in electrophoresis patterns, was selected for analysis. The glutenin fraction was extracted from the wheat flour and bread used as raw materials, and a positive correlation was observed when comparing the stable isotope ratios. It was found that the stable isotope ratio of the glutenin fraction reflects the characteristics of the stable isotope ratio derived from wheat. Therefore, bread was baked using wheat flour produced in Japan, the United States, and Canada, and the carbon and nitrogen isotope ratios of the glutenin fraction were compared. Japanese flour showed a tendency toward lower carbon and nitrogen isotope ratios compared to American and Canadian flours. This characteristic followed a similar trend to the carbon and nitrogen isotope ratios of the wheat flour itself. These results suggest that stable isotope ratio analysis of the gluten fraction could potentially be used to identify the origin of the wheat used as raw material in bread.

The seasonings used in this study are sugar, soy sauce, mirin and sake, all of which contain low levels of protein. Due to the seasoning’s penetrating effect, even if they contain a lot of water-soluble ingredients, the seasoning would remain in the meat tissue despite washing with water. This effect can be reduced by extracting the myofibrillar protein fraction after washing during the extraction process of the water-soluble fractions. If solids such as pepper are physically mixed into the cooked product, it is difficult to separate the meat, meaning washing with water alone is ineffective. Therefore, it is necessary to target protein fractions that are minimally affected by seasonings through sequential protein extraction.

### 4.2. δ^13^C, δ^15^N and δ^18^O Values of the Extracted Proteins from Japanese, the U.S., and Australian Beef

The carbon, nitrogen and oxygen isotope ratios of beef reflect the feeding and drinking water in the fattening environment and can be a useful tool for tracing the geographical origin of beef. Our results were similar to those reported by Nakashita et al. (2008) in previous studies [5]. The δ^13^C values of beef from the U.S. were −12.3 ± 1.1‰, which was significantly higher than those from Japan (−18.5 ± 1.0‰) and Australia (−22.5 ± 1.5‰). The δ^18^O values of beef from Australia (+16.0 ± 1.5‰) were significantly higher than in Japan (+10.9 ± 2.1‰) and the U.S. (+10.9 ± 1.1‰).

The PC1 in PCA is primarily influenced by oxygen isotope ratios. The oxygen isotope ratio of beef primarily reflects that of drinking water. Water oxygen isotope ratios have been reported worldwide. For example, Katsuyama et al. 2015 reported an oxygen isotope ratio map for Japan, showing values ranging from −13 to −6‰ [20]. Gabriel J. Bowen et al. reported a map of oxygen isotope ratios in tap water across the U.S. This map showed a wide range of values (−18 to −1‰) in beef-producing states, including Nebraska, Texas, Kansas, Iowa, and Colorado [21]. McInerney et al. 2023, presented a map of oxygen isotope ratios in Australian rainwater [22]. The primary beef cattle production regions of New South Wales, Queensland, and Victoria showed values ranging from −9 to −4‰. Notably, Australian beef imported by Japan originates from New South Wales, exhibiting relatively high values of −6 to −4‰. A comparison of literature values shows that the oxygen isotope ratios of water in the production areas of Australian beef exported to Japan are higher than those in beef production areas in Japan and the U.S. Therefore, the oxygen isotope ratios of water in each country are reflected in the oxygen isotope ratios of beef and it is considered that each country will exhibit characteristic values.

The PC1 in PCA is also influenced by nitrogen isotope ratios. The quality of beef depends significantly on the type of feed consumed by cattle. In particular, the balance between roughage and concentrate feed, as well as the type of grain provided, affects the flavor of the meat. The nitrogen isotope ratio of beef reflects that of the feed because cattle are herbivores and thus primary consumers in the trophic level. The nitrogen isotope ratio of feed reflects that of the nitrogen source in the soil absorbed by plant roots. Generally, organically grown plants have a higher nitrogen isotope ratio than plants grown with chemical fertilizers. Leguminous plants have lower nitrogen isotope ratios than other plants that depend on soil nitrogen, as they fix atmospheric nitrogen through symbiosis with rhizobia. Using leguminous plants such as soybeans in feed may decrease the nitrogen isotope ratio. Therefore, the ratio reflects the composition of the feed used for finishing.

The PC2 in PCA is influenced by carbon isotope ratios and primarily reflects differences in the proportion of C_4_ plants in the cattle feed. The carbon isotope ratios in animal tissue are mainly affected by the type of feed consumed. As each country’s cattle feed has its own characteristics, the stable isotope ratios in beef also differ accordingly.

The U.S. beef showed significantly higher carbon isotope ratios. The primary beef cattle production regions in the U.S. are Nebraska, Texas, Kansas, Iowa, and Colorado, accounting for approximately 70% of total production [23]. These states and their surrounding areas are major corn-producing regions in the U.S., enabling the inexpensive local procurement of corn, which constitutes the bulk of the concentrate feed for beef cattle. Nebraska, in particular, is located in the Corn Belt, a region that produces around 80% of the U.S. corn and 30% of the world’s corn. It is also a major beef-producing state. Corn is a C_4_ plant with a high carbon isotope ratio, which is thought to contribute to the high carbon isotope ratio of the U.S. beef.

In Australian beef production, grazing on large areas of land is an important factor. The Australian cattle reported by Nakashita et al. (2008) were also pasture-fed and showed a tendency towards low carbon isotope ratios [5]. However, frequent droughts in recent years have worsened pasture growth conditions, making them unstable. Consequently, grain-fed cattle raised in feedlots have also become important. Furthermore, demand for Australian grain-fed beef is rising, as is production, partly because major markets like Japan, South Korea, and China prefer marbled, juicy beef. Exports of grain-fed beef reached a record high of 121,157 tons, a 7% increase from the previous quarter, accounting for 29% of total beef exports [24]. Therefore, as the Australian cattle population includes both grass-fed and grain-fed cattle, the range of carbon isotope ratios is considered to be wide.

Beef cattle raised in Japan, including Wagyu and Holstein breeds, are generally not fed much grass in order to prevent the fat in their meat from turning yellow. To produce tender, marbled meat, they are not grazed but are raised in groups of Japanese Black cattle and fattened under strict control. Japanese Black cattle are generally fed a concentrated diet made from powdered or pressed corn, soybeans, wheat, bran, and other ingredients [25]. From 18 months of age until slaughter, they are fed a diet consisting of approximately 85% concentrated feed and 15.5% roughage, such as beer bran, hay, and rice straw. As the color of the meat is not a concern for dairy cows, many are pastured to graze grass, resulting in beef that often exhibits low carbon isotope ratios. Recently, more farms have been refattening cows for sale as table meat and feeding them a mixed diet of roughage and concentrated feed. As feeding management varies by farm, the carbon isotope ratios of Japanese beef are thought to exhibit a wide range of values.

## 5. Conclusions

We analyzed the stable isotope ratios of protein fractions extracted from defatted raw beef and cooked beef products to determine their geographical origin. The whole defatted simmered and marinated beef samples had significantly higher carbon and oxygen isotope ratios than the defatted whole raw beef due to seasonings such as soy sauce and sugar. In contrast, no significant difference was observed in the δ^13^C, δ^15^N, and δ^18^O values of proteins extracted from the defatted raw and cooked beef samples. The δ^13^C, δ^15^N, and δ^18^O value of the protein fraction extracted from beef showed characteristic distributions in Australia, Japan, and the U.S. Therefore, analysis of the δ^13^C, δ^15^N, and δ^18^O values of extracted proteins may be an effective way of determining the geographical origin of seasoned cooked beef products.

In this study, we proposed using the analysis of stable isotope ratios of myofibrillar protein fractions to determine the origin of beef samples that had been cooked with seasonings. However, the following points need to be examined further in future studies. Firstly, the seasoning used in this study had a low protein content. Highly processed foods such as hamburgers contain auxiliary ingredients with a high protein content, such as flour and eggs. The extraction procedure for these samples needs to be improved. Secondly, the number of beef samples collected from each country in this study was insufficient for tracing geographical origin, and an authentic database could not be established due to insufficient sample size. A statistical investigation using a larger number of samples will be conducted in the future, and annual variations in the stable isotope ratios of beef samples will be evaluated.

## Figures and Tables

**Table 1:** δ^13^C, δ^15^N and δ^18^O values of defatted whole beef samples and protein fractions in raw beef, boiled beef, marinated beef, and simmered beef.

	δ^13^C (‰)				δ^15^N (‰)				δ^18^O (‰)			
	Defatted Whole Sample		Protein		Defatted Whole Sample		Protein		Defatted Whole Sample		Protein	
	Average	1 σ	Average	1 σ	Average	1 σ	Average	1 σ	Average	1 σ	Average	1 σ
Raw beef	−21.2	0.0	−21.1	0.0	6.9	0.0	7.1	0.0	15.9	0.3	16.4	0.3
Boiled beef	−21.3	0.1	−21.1	0.0	6.5	0.2	7.0	0.1	14.4	0.1	16.2	0.2
Marinated beef	−20.6	0.0	−21.1	0.1	6.9	0.1	7.0	0.0	22.6	0.4	16.5	0.3
Simmered beef	−19.8	0.2	−21.1	0.0	6.4	0.1	7.1	0.1	27.4	0.4	16.3	0.4

## Data Availability

The dataset presented in this paper is not readily available due to its potential use in inspection testing. For inquiries regarding access to the dataset, please contact the corresponding author directly.

## Abbreviations

The following abbreviations are used in this manuscript:

GIs	Geographical indications
CHAPS	(3-(3-Cholamidopropyl)dimethylammonio)-1-propanesulfonate
EA/IRMS	Elemental analyzer/isotope ratio mass spectrometer
IAEA	International Atomic Energy Agency
NIST	National Institute of Standards and Technology
ANOVA	Analysis of variance
PCA	Principal component analysis
SDS-PAGE	SDS-polyacrylamide gel electrophoresis
